# NKp44-Derived Peptide Binds Proliferating Cell Nuclear Antigen and Mediates Tumor Cell Death

**DOI:** 10.3389/fimmu.2018.01114

**Published:** 2018-05-23

**Authors:** Avishai Shemesh, Kiran Kundu, Refael Peleg, Rami Yossef, Irena Kaplanov, Susmita Ghosh, Yana Khrapunsky, Orly Gershoni-Yahalom, Tatiana Rabinski, Adelheid Cerwenka, Roee Atlas, Angel Porgador

**Affiliations:** ^1^The Shraga Segal Department of Microbiology, Immunology and Genetics, Faculty of Health Sciences, Ben-Gurion University of the Negev, Beer Sheva, Israel; ^2^National Institute for Biotechnology in the Negev, Ben-Gurion University of the Negev, Beer Sheva, Israel; ^3^Surgery Branch, National Cancer Institute, National Institutes of Health, Bethesda, MD, United States; ^4^Innate Immunity Group, German Cancer Research Center and Medical Faculty Mannheim, Heidelberg University, Heidelberg, Germany

**Keywords:** NKp44, peptide screen, cell-penetrating peptide, proliferating cell nuclear antigen, cancer therapy

## Abstract

Proliferating cell nuclear antigen (PCNA) is considered as a hub protein and is a key regulator of DNA replication, repair, cell cycle control, and apoptosis. PCNA is overexpressed in many cancer types, and PCNA overexpression is correlated with cancer virulence. Membrane-associated PCNA is a ligand for the NKp44 (NCR2) innate immune receptor. The purpose of this study was to characterize the PCNA-binding site within NKp44. We have identified NKp44-derived linear peptide (pep8), which can specifically interact with PCNA and partly block the NKp44–PCNA interaction. We then tested whether NKp44-derived pep8 (NKp44-pep8) fused to cell-penetrating peptides (CPPs) can be employed for targeting the intracellular PCNA for the purpose of anticancer therapy. Treatment of tumor cells with NKp44-pep8, fused to R11-NLS cell-penetrating peptide (R11-NLS-pep8), reduced cell viability and promoted cell death, in various murine and human cancer cell lines. Administration of R11-NLS-pep8 to tumor-bearing mice suppressed tumor growth in the 4T1 breast cancer and the B16 melanoma *in vivo* models. We therefore identified the NKp44 binding site to PCNA and further developed an NKp44-peptide-based agent that can inhibit tumor growth through interfering with the function of intracellular PCNA in the tumor cell.

## Introduction

NKp44 is a dual functional receptor, manifesting activation and inhibitory phenotype that was first characterized on natural killer (NK) cells (1–3). NKp44 recognizes proliferating cell nuclear antigen (PCNA) expressed on the membrane of cancer cells (4–10). PCNA recognized by NKp44 can lead to inhibition of NK cell functions (11, 12).

Proliferating cell nuclear antigen is a hub protein that is greatly conserved among mammals and can interact with many proteins (13–16). PCNA intracellular functional range is associated with DNA replication, DNA repair, cell cycle control, apoptosis, chromatin metabolism, and gene expression (17–19). PCNA expression is upregulated in cancer cells relative to healthy normal cells and can be used as a marker of cell proliferation and cancer virulence in many types of cancers (9, 20–27). Therefore, PCNA is considered as a potential therapeutic target in anticancer therapy (28, 29).

Targeting PCNA *via* peptides was shown to have a profound impact on cancer cells growth (29, 30). These peptides are derived either from functional binding domains within PCNA or from conserved binding motifs, found within the protein ligands of PCNA (29). The first peptides group contains the caPep peptide and the Y211F-based peptides, which are derived from the L126-Y133 PCNA sequence and the proximal region of Y211-PCNA, respectively (31–33). The caPep peptide blocks the interaction of intracellular proteins to PCNA in the interdomain connecting loop (IDCL, L118-C135) domain, while Y211F-based peptides inhibit PCNA tyrosine phosphorylation (Y211), mediated by EGFR and thus block PCNA interaction to c-Abl (34). The second peptides group contains (i) PCNA-interacting peptide (PIP) box-based peptides (QxxL/I/Mx xHF/DF/Y), and (ii) APIM-based peptides (R/K–F/W/Y–L/I/V/A–L/I/V/A–K/R), which are peptides derived from sequences of intracellular proteins interacting with PCNA (35–39). PIP-based peptides and APIM-based peptides interaction with PCNA involve the IDCL domain on PCNA, and consequently block PCNA interactions with its target proteins. PCNA-targeting peptides were shown to inhibit the growth or to induce apoptosis in neuroblastoma, hormone-insensitive prostate cancer, triple-negative breast cancer, bladder cancer, and multiple myeloma (31, 32, 38, 40, 41).

Since NKp44 interact with PCNA, we hypothesized that NKp44-derived linear peptides could specifically bind PCNA and lead to inhibition of cancer cell proliferation and/or lead to cell death. Therefore, we screened NKp44-derived successive peptides, 20 amino acid long, for binding to PCNA and blocking of NKp44–PCNA interaction. We then examined the potential of identified PCNA-binding NKp44-derived peptides, conjugated to cell-penetrating moieties, to (i) inhibit cancer cell proliferation or induce apoptosis *in vitro* and (ii) mediate tumor growth arrest *in vivo*, in several mouse and human cancer cell lines. We characterized one potential peptide, NKp44-derived pep8 (NKp44-pep8), which manifested a specific interaction with PCNA and blocked NKp44 binding to PCNA; upon fusion to cell-penetrating peptide (CPP), pep8 induced tumor cell death *in vitro* as well as tumor growth suppression *in vivo*. These results demonstrate that NKp44-pep8 take part in the binding site of PCNA on NKp44 and it is a potential tool for anticancer therapy.

## Materials and Methods

### Peptides Synthesis

NKp44-derived peptides were synthesized with/without biotin using PEP screen technology, which is a peptide synthesis platform that utilizes Fmoc protective chemistry (Sigma-Aldrich). Nineteen overlapping peptides (20mer peptides with a 15-aa overlap between successive peptides) covered the NKp44ectodomain and hinge region (amino acids: 22–131; Accession: CAB39168.1; www.ncbi.nlm.nih.gov) were synthesized. Single nucleotide polymorphism (NCBI; SNP; rs9471577) lead to a replacement of M → V in amino acid #75 on NKp44, and we used this valine replacement in the synthesized peptides (in peptides 8–10). Selected peptides and CPP containing peptides were ordered from GL Biochem (Shanghai, China, purity > 95%, in TFA salt and acetate salt for *in vitro* and *in vivo* assays, respectively). Lyophilized peptide stocks were kept frozen in dehydrating conditions. Stock solutions of peptides (2 mM) were solubilized in DDW–5% DMSO and stored in frozen aliquots. The following CPPs were used to test the function of NKp44-pep8; miniAntp (KRRMKWKK), SV40 large T antigen NLS (PKKKRRV), Transferrin receptor binding peptide (TfR) (HAIYPRH), R9 (RRRRRRRRR), or R11 (RRRRRRRRRRR) (42–48).

### Cell Lines

Following murine cell lines: 4T1; mammary carcinoma (ATCC^®^ CRL-2539™), B16-F0; melanoma (ATCC^®^ CRL-6322™) and human cell lines: A549; lung carcinoma (ATCC^®^ CCL-185™), MDA-MB-23; breast adenocarcinoma (ATCC^®^ HTB-26™), HepG2; hepatocellular carcinoma (ATCC^®^ HB-8065™), PANC-1; pancreas ductal adenocarcinoma (ATCC^®^ CRL-1469™) were used in this study. Culture medium was prepared as following; DMEM (Gibco, 41965-039) supplemented with 10% fetal calf serum (FCS) (Gibco, 12657-029), 1% l-glutamine (BI, 03-020-1A), 1% Pen–Strep (BI, 03-031-1B), 1% sodium pyruvate (BI, 03-042-1B), 1% MEM-Eagle (BI, 01-340-1B), and 1% HEPES 1 M (BI, 03-025-1B). NK92-44-1 cells were cultured as previously described (11, 12).

### Mice Strains

Six- to eight-week-old C57BL/6 male and BALB/C female mice were purchased from Envigo/Harlan Laboratories (Rehovot, Israel). Maintenance and breading of all mice used in this study were done in the local animal care facility, approved by the Institutional Animal Care and Use Committee of Ben-Gurion University of the Negev. Revision and approval of all experimental procedures were done by the Institutional Animal Care and Use Committee of Ben-Gurion University of the Negev (BGU’s IACUC) according to specified protocols that aim to ensure animal welfare and reduce suffering (permit: 31.35.13).

### Recombinant His-Tag and MBP-Fusion Proteins Production

The pET-28 or pMAL-c2x vectors were used to produce soluble human PCNA (hPCNA) in Rosetta™ 2 (DE3) cells. Plasmids containing the mRNA sequence of PCNA or TL1A, APO-E3, HNF4 were transformed into Rosetta™ 2 cells *via* heat shock and grown on LB agar plates with kanamycin and chloramphenicol. A fresh colony of transformed bacteria was grown overnight in a 5 ml of LB with kanamycin and chloramphenicol an incubator shaker set to 37°C and at 250 rpm. The next day, bacteria cells were diluted 1:100 into a 500 ml of LB with kanamycin and chloramphenicol (to discard dead cells) and grown to an optical density (O.D.) of 0.6–0.8 (λ = 650 nm). Isopropyl β-d-1-thiogalactopyranoside (0.5 mM) was added for the induction of the PCNA production, and cells were further incubated for 4 h at 26°C (hPCNA). Cells were then centrifuged and the pellet was resuspended with solution A (20 mM Tris–HCl pH 8.0, 0.5 M NaCl, 20 mM imidazole), sonicated six times (20 s with 40 s intervals) and sieved through 0.45 µM (Sartorius Stedim Biotech) filter before loaded on a His-tag or Amylose resin beads. Purification of His-tagged proteins was done using a Gravity-flow Column with “HisPur™ Ni-NTA Resin” Bead kit (Thermo-Scientific) (binding capacity ≤ 60 mg of a 28 kDa 6xHis-tagged protein from a bacterial source per milliliter of settled resin) according to Kits Protocol. Purification of MBP-tagged proteins was done using the Amylose resin beads (Catalog # E8035S).

### ELISA Assays

To determine the interaction between NKp44-derived peptides and PCNA, ELISA plates were coated overnight at 4°C with 2 µg/ml of the recombinant PCNA or TLA1 (49), APO-E3 (50), HNF4 (4), rhFc, rhNKp44-hFc (12), rhNKp46-hFc (51), and PBS 1× (negative controls). Blocking buffer (PBS + 2.5% skim milk) was applied for 2 h at room temperature, after that the plates were incubated with 0–8 µM of biotinylated NKp44-derived peptides or PBS 1× (background control) for 2 h at room temperature. NKp44-derived peptides binding to PCNA was detected using horseradish peroxidase (HRP)-conjugated to streptavidin (1 h at 1:500 dilution). Following washing, TMB (DAKO, S1599) was added. O.D. was read at 650 nm (Thermo Electron Corporation Multiskan Spectrum). Between each step, wells were washed three times with PBS containing 0.05% Tween 20 (PBST).

To determine the ability of NKp44-pep8 to block NKp44-hFc interaction with PCNA, ELISA plates were coated overnight at 4°C with 2 µg/ml of the recombinant PCNA, blocking buffer (PBS + 2.5% skim milk) was applied for 2 h at room temperature, after which the plates were incubated with 0–10 µM of biotinylated NKp44-derived peptides or PBS 1× (100 μl/well, background control) for 2 h at room temperature and then NKp44-hFc (80 nM, 20 µl/well) was added without washing for 1 h at room temperature. NKp44-hFc binding to PCNA was detected using HRP conjugated to antihuman IgG (1 h at 1:400 dilution 100 μl/well). Following washing, TMB (100 μl/well DAKO, S1599) was added. O.D. was read at 650 nm (Thermo Electron Corporation Multiskan Spectrum). Between each step, wells were washed three times with 200 μl/well PBS 1× containing 0.05% Tween 20 (PBST).

To determine the ability of APIM-based peptide to suppress NKp44-pep8 interaction with PCNA, ELISA plates were coated with recombinant PCNA followed by PBS–skim milk added as described earlier. Plates were then incubated with 25 µg/ml of R11-NLS-pep8 (positive control), R11-NLS-pep8short (negative control), and APIM for 1 h at room temperature followed by addition of biotin-pep8 (5 µg/ml) without washing for 1 h at room temperature. Biotin-pep8 binding to PCNA was detected using streptavidin conjugated HRP (1 h at 1:500 dilution 100 μl/well). Following washing, TMB (100 μl/well DAKO, S1599) was added. O.D. was read at 650 nm (Thermo Electron Corporation Multiskan Spectrum).

IFNγ secretion assay and ELISA assay were done as previously described (11, 12). NKp44-pep8, pep9, and pep10 (10 or 20 µg) were added to the IFNγ secretion assay.

### Kinetic Analysis by Surface Plasmon Resonance (SPR)

A ProteOn™ XPR36 Protein Interaction Array System (Bio-Rad) was used to measure the affinity of NKp44-derived peptides to MBP-fused recombinant hPCNA. For the assay, a GLC sensor chip (7CG21401) and ProteOn Manager Version 3.1.0.6 (Bio-Rad Laboratories) was employed. After activation of the chip using EDC/S-NHS amine coupling procedure, the ligand immobilization process was performed with NKp44-pep8, R11-NLS-pep8, R11-NLS-pep8short, and BSA as a control at a flow rate of 30 µl/min in different flow cells. Different concentrations (0–250 nM) of analyte (MBP-PCNA) were then injected at a flow rate of 25 µl/min. Regeneration of the surface was done using 50 mM NaOH. Data were analyzed using equilibrium analysis model.

### Cell Viability Assay Employing PrestoBlue

Target cells (2.5 × 10^4^ cells/well of a 48-well plate, Corning) were seeded and incubated in 37°C, 5% CO_2_ for 24 h in 0.5 ml of fresh complete DMEM (10% FBS) culture medium. According to molecular weight peptides were diluted from 8 to 2 µM with 5% FBS containing DMEM culture medium just before the incubation with the target cells. Medium was removed from each well, and 250 µl of diluted peptides was added and incubated for another 24 h at 37°C, 5% CO_2_. PrestoBlue (A13261, Invitrogen™) solution was prepared by diluting the dye 10-fold with complete DMEM. Medium was discarded from each well, and 250 µl of PrestoBlue solution was added to each well, shake gently and incubated at 37°C for another 1 h. Emission at 560 nm was measured in Premium Quad4 Monochromators plate reader (Tecan). Normalization was done relative to the appropriate control (DMEM or DMSO) to calculate the percentage of viable cells in each group. Selenite (25 µM) was used as a positive control (52, 53).

### Flow Cytometry Based Cell Death Assay

Peptides were diluted before the incubation with the target cells with free DMEM culture medium to 0–16 µM according to peptide molecular weight. For adding the peptides, 0.5 ml was removed from each well, and 0.5 ml of diluted peptides was added to a final volume of 1 ml, 5% FCS, duplicates. Peptides were incubated with target cells for 24 h, and then the medium was collected, adherent cells were removed with trypsin–EDTA (0.5 ml, 3 min, BI: 03-052-1B), 1 ml of complete medium was added to each well, and the medium was collected (final volume 2.5 ml, on ice). For the cell death assay, samples were then centrifuged (1,300 rpm, 4°C, 5 min), the medium was removed, fresh 1 ml 10% FCS was added, and then 1 µl of 1 mg/ml PI was added to each sample and incubated (15 min, on ice) with the target cells. Before analyzing the samples, samples were centrifuged (1,300 rpm, 4°C, 5 min), the medium was removed, and fresh 0.2 ml FCAS buffer (PBS 1×, 20% FCS, 0.5% NaN_3_) was added. Samples were then read and analyzed using BD FACSCanto™ II and FlowJo^®^. APIM peptide and camptothecin (CPT) were used as positive controls (38, 54–56). Specific cell death was calculated as following: *Y* = 100*((sample cell death − basal cell death)/(100 − basal cell death)). Basal cell death = % of PI positive cells following incubation in complete culture media.

### *In Vivo* Tumor Growth and Treatments

Seven-week-old female BALB/C mice with an average body weight of 20 g were used in this experiment. 4T1 cells suspended at a density of 2 × 10^5^ cells/50 μl, in serum-free DMEM containing 40% Geltrex, were injected into the left mammary fat pad, and allowed to develop tumors. After 5 days, mice were randomly divided into three groups, one group was treated with R11-NLS-pep8, at a dose of 5 mg/kg, intravenously (IV), other group with 5-FU, at a dose of 30 mg/kg, (IV) and mice treated with a 1.44% DMSO/150 mM NaCl solution, (IV) served as the vehicle control. Treatment was continued following every 2 days at least for 4 weeks. Each week, tumor size was evaluated by measuring their length (*L*) and width (*W*), using a Caliper device and tumor volume (*V*) was calculated according to the equation: *V* = *LW*^2^/2. After 4 weeks of treatment, mice were euthanized, and tumors volume and weight were measured.

Seven-week-old male C57BL/6 mice with an average body weight of 23 g were used in the other experiment. B16 cells suspended at a density of 0.5 × 10^6^ cells/100 μl, in serum-free DMEM containing 40% Geltrex, were injected subcutaneously in the left dorsal flank, and allowed to develop tumors. After 6 days, mice were randomly divided into three groups, one group was treated with R11-NLS-pep8, at a dose of 5 mg/kg, intraperitoneally (IP), other group with 5-FU, at a dose of 30 mg/kg, (IP) and mice treated with a 1.44% DMSO/150 mM NaCl solution, (IP) served as the vehicle control. Treatment was done three times a week at least for 4 weeks. Each week, tumors size was evaluated by measuring their length (*L*) and width (*W*), using a Caliper device and tumor volume (*V*) was calculated according to the equation: *V* = *LW*^2^/2. After 3 weeks of treatment, mice were euthanized, and tumors volume and weight were measured.

### Statistics

Graphics and statistical analysis were performed using GraphPad Prism software. Statistical analysis of the data was performed using unpaired *t*-test (with *p*-values of *p < 0.05, **p < 0.01 or ***p < 0.001, ****p < 0.0001 as indicated on the figures).

## Results

### NKp44-pep8 Binds to Recombinant PCNA and Inhibit NKp44 Binding

**Figure 1:**
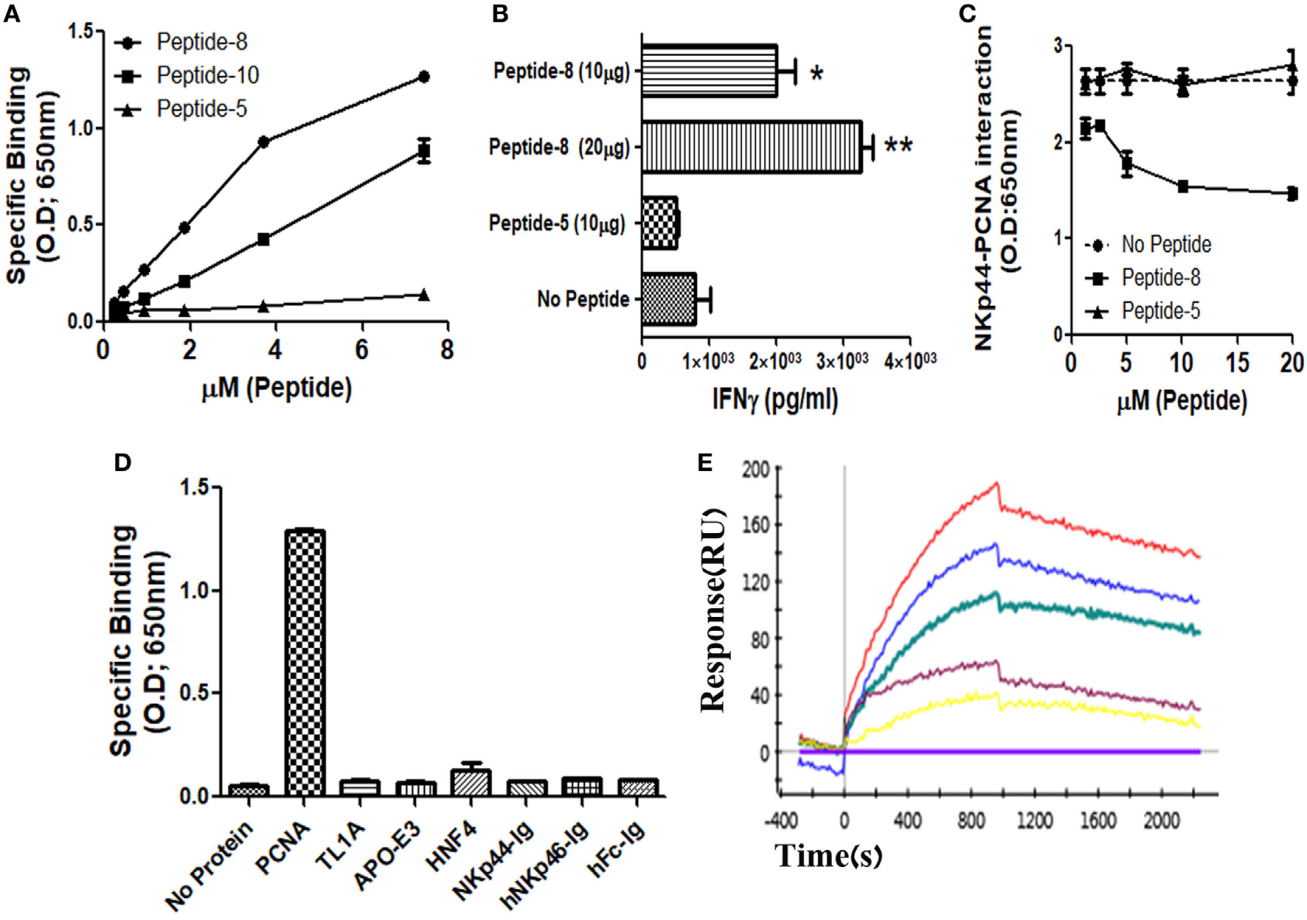
Recognition of recombinant human PCNA (hPCNA) by NKp44-derived pep8 (NKp44-pep8). **(A)** ELISA assay representing the binding curve of biotinylated NKp44-derived pep5, pep8, and pep10 at a peptide concentration range of 0–7.24 µM to plate bond hPCNA. **(B)** IFNγ secretion assay of NK92-44-1 cells co-incubate with the HLA positive cell line PANC-1 (E:T ration of 1:2) in the presence of NKp44-pep8 (10 or 20 μg/well), NKp44-derived pep5 was used as negative control. Note that when we apply to NK92-44-1 pep8 (without target cells), activation of IFNγ secretion is minimal and less/similar to the observed activation when applying the pep5 negative control. **(C)** ELISA assay of blocking NKp44–proliferating cell nuclear antigen (PCNA) interaction *via* NKp44-pep8. NKp44-derived pep5 was used as a negative control. No peptide represents the maximal binding capacity of NKp44 to hPCNA. **(D)** ELISA assay showing the specific recognition of NKp44-pep8 (5 µg/ml) to hPCNA relative to TL1A, APO-E3, HNF4, NKp44-Ig, hNKp46-Ig, hFc. **(E)** ProteOn array showing the binding of hPCNA at protein concentration range of 0–250 nM to bond NKp44-pep8. 0 nM (X base-line), 15.6 nM (yellow), 31.2 (purple), 62.5 nM (green), 125 nM (blue), and 250 nM (red).

We and others previously showed that membrane-associated PCNA is a ligand for NKp44 (4, 7, 12). In this study, we aimed to identify the PCNA-binding NKp44 site by dividing NKp44 ectodomain and hinge region into 19 overlapping, 20mer peptides with a 15-aa overlap between successive peptides. We first studied whether these 19 peptides could bind to recombinant PCNA protein. Among the 19-screened peptides, NKp44-derived peptide #8 (pep8) manifested a substantial binding to PCNA, while overlapping NKp44-derived peptide #10 (pep10) also bound to PCNA but to a lesser extent as compared with pep8. Table 1 shows the pep8 and pep10 amino acid sequences. Figure 1A shows the binding of titrated concentrations of pep8 and pep10 to recombinant hPCNA as compared with the negative binding of NKp44-derived peptide #5 (pep5), which is representative of the negative binding of the other screened NKp44-derived peptides. The 10-mer shared core sequence of pep8 and pep10 did not bind PCNA (data not shown), possibly due to conformational stability or due to 3D peptide structure that did not fit to the structure of the PCNA-binding motif in the NKp44 protein ectodomain. Co-incubation of NK92 cells overexpressing NKp44-1 splice variant, with the HLA-I positive, PANC-1 cells, in the presence of pep8, led to increase in the secretion of IFNγ (Figure 1B). pep8 was capable of mediating inhibition of rhNKp44 binding to PCNA; Figure 1C shows the inhibition of rhNKp44 binding to PCNA for titrated amounts of pep8 as compared with negative control pep5. Binding of pep8 to PCNA was specific, as pep8 did not bind other recombinant proteins, produced similarly to PCNA, such as TL1A, APO-E3 and HNF4 (Figure 1D). We previously published that NKp46-drived peptide #4 inhibits NKp46 function through the binding to NKp46 itself (57). However, NKp44-pep8 did not bind to rhNKp44 (Figure 1D). Therefore, the inhibition of NKp44–PCNA interaction was solely due to its interaction of pep8 with PCNA. To test the affinity of this interaction we employ kinetic analysis by SPR to check the affinity of pep8 to PCNA. pep8 displayed a characteristic binding curve to PCNA (*K*_D_ = 4.9E−07 M), indicating binding with a very moderate affinity (Figure 1E). Taken together, these results indicate that pep8 domain on NKp44 takes part in the NKp44–PCNA recognition and that pep8 specifically interact with PCNA.

**Table 1:** Amino acid sequences of peptides employed in this study.

Name	M.W. (Da)	Sequence	Modifications
**NKp44-derived peptides^a^**			
pep8	2,101.49	EASALVCIRLVTSSKPRTVA	Biotin or purified (N-terminal)
pep8short	1,045.2	VTSSKPRTVA	
pep10	2,265.60	VTSSKPRTVAWTSRFTIWAA	
pep9	2,307.74	VCIRLVTSSKPRTVAWTSRF	
pep5	2,228.55	YPPTGSLYEKKGWCKEASAL	

**NKp44-derived pep8-cell-penetrating peptide chimeras**			
Mini-Antp-pep8	3,243.97	KRRMKWKK-EASALVCIRLVTSSKPRTVA	Ac (N-terminal), D isomer last amino acid
NLS-pep8	2,994.64	PKKKRRV-EASALVCIRLVTSSKPRTVA	
Transferin-pep8	2,976.49	HAIYPRH-EASALVCIRLVTSSKPRTVA	
Mini-Antp/NLS-pep8	3,656.51	KRRMKW-KKKRK-EASALVCIRLVTSSKPRTVA	
Transferrin-NLS-pep8	3,645.38	HAIYPRH-KKKRK-EASALVCIRLVTSSKPRTVA	
TransferinTransferin-pep8	3,851.49	HAIYPR-HHAIYPR-HEASALVCIRLVTSSKPRTVA	
R9-NLS-pep8	4,176.06	RRRRRRRRR-KKKRK-EASALVCIRLVTSSKPRTVA	
R11-NLS-pep8	4,787.81	RRRRRRRRRRR-I-KKKRK-W-EASALVCIRLVTSSKPRTVA	
pep8-NLS-R11	4,787.81	EASALVCIRLVTSSKPRTVA-W-KKKRK-I-RRRRRRRRRRR	
R11-NLS-pep8short	3,731.52	RRRRRRRRRRR-I-KKKRK-W-VTSSKPRTVA	

**Published PCNA-proliferating cell nuclear antigen-targeting peptides**			
APIM	3,633.48	MDRWLVK-W-KKKRK-I-RRRRRRRRRRR	Ac (N-terminal), D isomer last amino acid

*^a^Amino acid sequences of NKp44-derived peptides that were not part of the figures are not shown*.

### NKp44-pep8, Conjugated to CPP, Reduces Cell Viability

**Figure 2:**
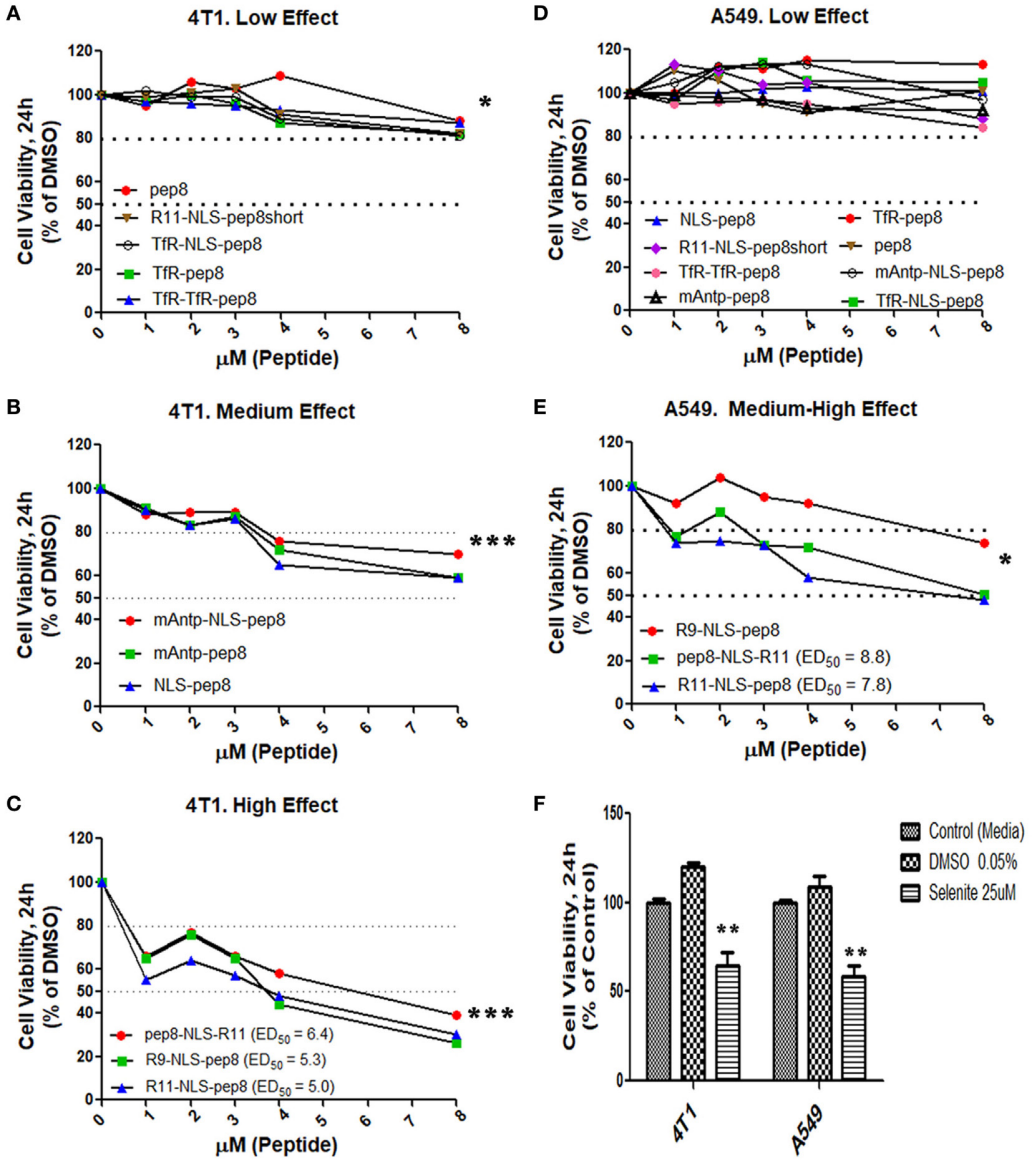
Cell viability screening of cell-penetrating peptides (CPPs) NKp44-derived pep8 (NKp44-pep8) chimeras using PrestoBlue based cell viability assay. Mouse 4T1 breast cancer and human A549 lung cancer cell lines were used for screening different CPP fused to NK44-derived pep8 and their effect on cell viability in a PrestoBlue assay. The results are shown in respect to the basal viability indicated using equivalent % DMSO (background). CPPs-NKp44-pep8 were divided according to their effect on cell viability and the cancer model. **(A)** 4T1: low effect (**p, group at 8 µM), **(B)** 4T1: medium effect (***p, group at 8 µM), **(C)** 4T1: high effect (***p, group at 8 µM), **(D)** A549: low effect, **(E)** A549: medium-high effect (*p, group at 8 µM), ED50 was calculated using a sigmoidal dose–response curve only for CPPs-NKp44-pep8 that have shown reduced viability below 50% after 24 h. **(F)** Cell viability of DMSO (negative control) or selenite (positive control) treated 4T1 and A549 cells relative to complete medium. Statistical analysis was performed in comparison to DMSO. Unpaired *t*-test; one-tail, *p < 0.05, **p < 0.01, and ***p < 0.001.

Disruption of intracellular PCNA activity affects tumor cell viability (31, 32, 38). We hypothesized that penetration of PCNA-binding NKp44-pep8 into the cell will result in inhibition of cell proliferation and/or lead to cell death. Therefore, we designed a number of combinations of CPPs fused to NKp44-pep8 (Table 1). Effects of CPP-pep8 combinations were tested on the mouse 4T1 breast cancer cells and on the human A549 lung adenocarcinoma cells (Figure 2). Both mouse and human tumor models were investigated since PCNA is a conserved protein with a high degree of homology across mammalian (16). We first assessed CPP-pep8 effect on cell viability employing the PrestoBlue reagent, in a peptide concentration range of 0–8 µM, applied to cells for 24 h. We defined low, medium, and high effect of CPP-pep8 on cell viability (down to 80, 50–80, and <50% viability, respectively) as compared with negative control. Since peptide stocks were solubilized in 5% DMSO, negative controls included both “medium only” and “medium with 0.005% DMSO,” reflecting percent DMSO in the highest concentration of applied peptide (8 µM). Figures 2A–C show the peptides manifested low (Figure 2A), medium (Figure 2B), and high effect (Figure 2C) on 4T1 cells. As expected, pep8 alone, without any CPP, is included in Figure 2A with almost no effect on cell viability. The TfR (with or w/o NLS) and TfR–TfR CPPs preceding pep8 manifested low effect on the viability of 4T1 cells, while the miniAntp (with or w/o NLS) and NLS CPPs preceding pep8 manifested medium effect. Only R11 or R9 CPPS preceding pep8 or R11 succeeding pep8 demonstrated high effect on the cell viability of 4T1 cells. The high effect was specific to pep8-PCNA interaction, since R11 CPP preceding the short form of pep8 (pep8short), which does not bind PCNA, did not affect 4T1 cell viability (Figure 2A).

A549 cells were less sensitive to CPP-pep8 effect as compared with 4T1. All combinations involving miniAntp and TfR/TfR–TfR CPPs manifested negative to low effect on cell viability of A549 cells (Figure 2D). As before, pep8 alone or CPP-pep8short did not show any effect on tumor cell viability. In accordance with 4T1, arginine-based CPPs showed the higher potency, with medium effect for R9-based CPP and high effect for R11-based CPP (Figure 2E). Figure 2F shows the controls employed in these experiments. Medium containing DMSO (0.005%, negative control) did not reduce cell viability. Selenite, the positive control significantly reduced cell viability, manifested similar suppressive effect on the cell viability of 4T1 and A549 cells, which resembled the medium effect of the CPP-pep8 peptides.

**Figure 3:**
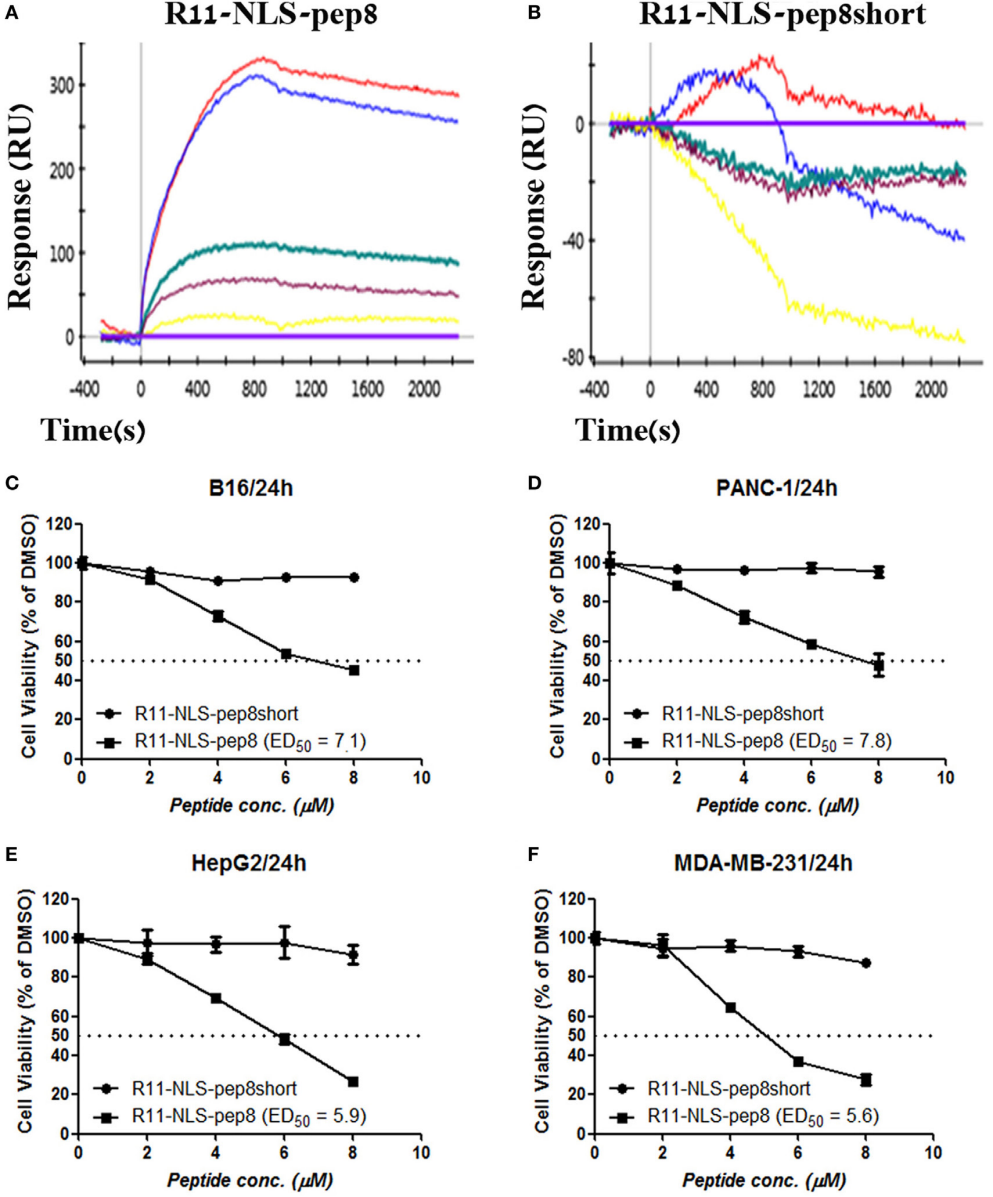
ProteOn based affinity array of R11-NLS-pep8 toward proliferating cell nuclear antigen (PCNA) and cell viability screening of human and murine cell lines. **(A,B)** ProteOn array result showing the binding of human PCNA at protein concentration range of; 0 nM (X base-line), 15.6 nM (yellow), 31.2 (purple), 62.5 nM (green), 125 nM (blue), 250 nM (red), to bond R11-NLS-pep8 and R11-NLS-pep8short, respectively. Murine B16 melanoma, human PANC-1 (pancreas ductal adenocarcinoma), HepG2 (liver hepatocellular carcinoma), and MDA-MB-231 (breast adenocarcinoma) cancer cell lines are used for checking the effect of R11-NLS-pep8 and R11-NLS-pep8short on cell viability in a PrestoBlue assay. The results are shown in respect to the basal viability indicated using equivalent % DMSO (background). ED50 was calculated using a sigmoidal dose–response curve. ED50 values were 7.1, 7.8, 5.9, and 5.6 µM, respectively, for B16, PANC-1, HepG2, and MDA-MB-231 **(C–F)**.

Since R11-NLS-pep8 showed the strongest reduction in PrestoBlue staining for both murine 4T1 and human A549 cell lines, we tested its binding affinity to PCNA and its functional efficacy on other murine and human tumor cell lines. R11-NLS-pep8 displayed a characteristic binding curve to PCNA (*K*_D_ = 8.7E−08 M), indicating binding with a moderate affinity (Figure 3A). This affinity was higher than pep8 binding affinity to PCNA (Figure 1E), however, in the same ballpark. Yet, binding was pep8 specific since affinity of R11-NLS-pep8short to PCNA was null (Figure 3B), as we observed before for pep8short. We further compared the cell viability effect of R11-NLS-pep8 and R11-NLS-pep8short on the mouse B16 melanoma, PANC-1—human pancreatic adenocarcinoma, HepG2—human hepatocellular carcinoma, and MDA-MB-231—human breast adenocarcinoma. R11-NLS-pep8short had none to dull effect on the cell viability of these cell lines, while R11-NLS-pep8 manifested high effect (as defined above) on the cell viability of these lines (Figures 3C–F).

### NKp44-pep8, Conjugated to R11-NLS CPP, Mediates Tumor Cell Death

**Figure 4:**
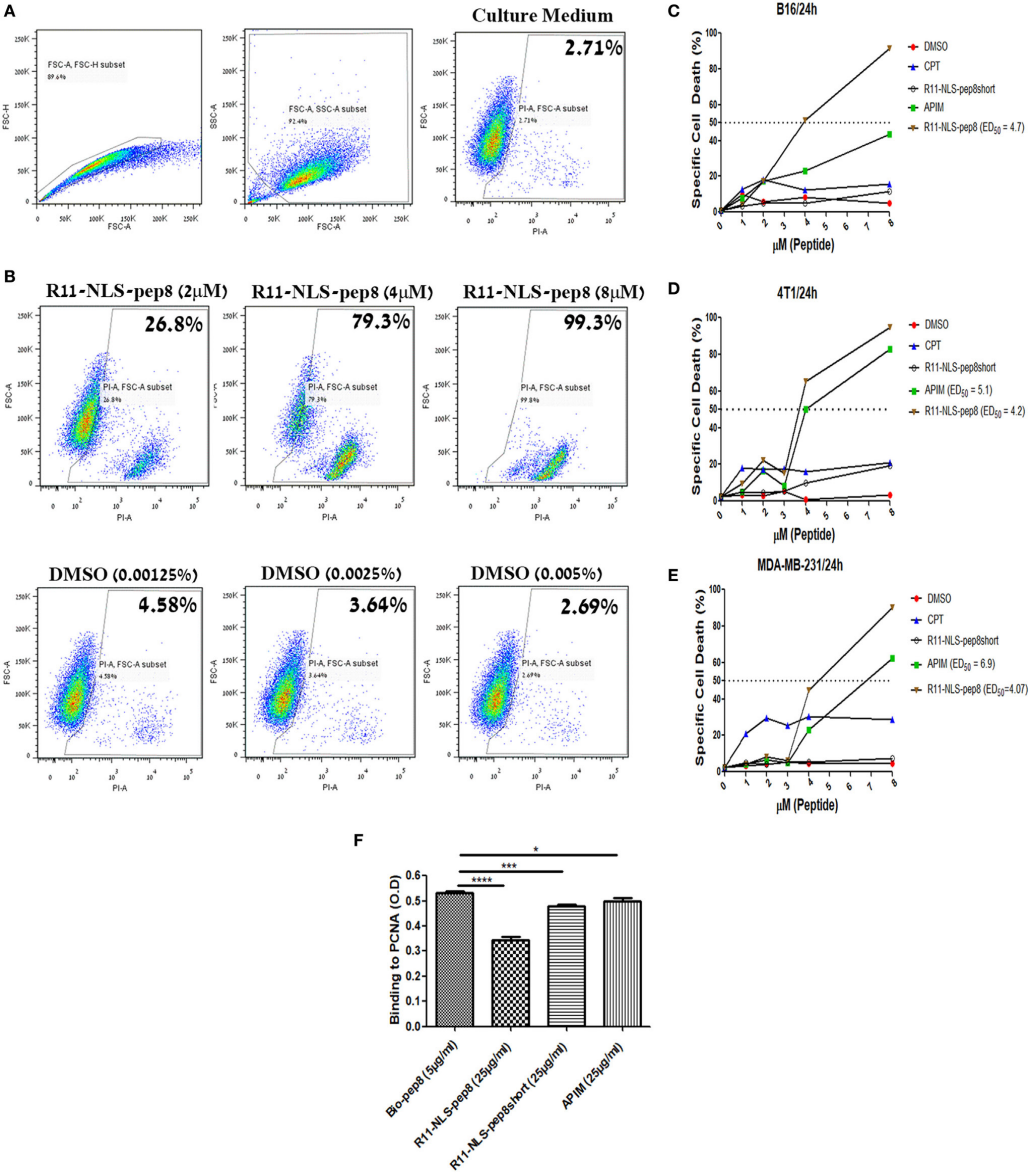
Flow cytometry based cell death assay of R11-NLS-pep8. Flow cytometry based cell death assay using PI was employed. **(A)** Gating strategy of cancer cell line for the detection of PI positive (dead) cells in culture medium, **(B)** representative dot plots for the effect of R11-NLS-pep8 on cell death relative to equivalent DMSO percentages. **(C)** Cell death assay of murine B16 cell line, **(D)** cell death assay of murine 4T1 cell line, **(E)** cell death assay of human MDA-MB-231 cell line. Camptothecin and APIM peptide were used as positive control; R11-NLS-pep8short was used as a negative control. ED50 was calculated using a sigmoidal dose–response curve only for treatments that have shown to induce cell death above 50% after 24 h. **(F)** ELISA-based assay to investigate pep8 binding site to proliferating cell nuclear antigen. R11-NLS-pep8 was employed as positive control and R11-NLS-pep8short served as a negative control. Unpaired *t*-test, *p < 0.05, **p < 0.01, and ***p < 0.001.

The role of PCNA extends over regulation of cell cycle, DNA synthesis, DNA repair and cellular death. However, we could not able to differentiate whether it is CPP-pep8 mediated reduction in cell proliferation or induction of cell death using PrestoBlue assay. To investigate the mechanism, we studied the effect of CPP-pep8 on cell death by flow cytometry analysis using the fluorescent cell death marker, PI. We focused on R11-NLS-pep8 since this CPP-pep8 showed the strongest reduction in PrestoBlue staining (Figures 2C,E). The cytotoxic agent, CPT, an inhibitor of topoisomerase I, and the published APIM peptide, which is interacting with intracellular PCNA (38, 54, 55), were used as positive controls. Peptide R11-NLS-pep8short was used as a negative control. Cells were incubated with each agent at a concentration range of 0–8 µM for 24 h and then assayed for cell death. R11-NLS-pep8 manifested a positive effect. Figure 4A shows the gating strategy used for the flow cytometry data analysis and representative results for incubating cells with control medium. Figure 4B shows representative results for incubating cells with titrated amounts of R11-NLS-pep8 and control DMSO representing the DMSO concentrations in the media for the respective R11-NLS-pep8 concentrations. Incubation of R11-NLS-pep8 for 24 h, with the murine cell lines B16 (C57BL/6 origin) and 4T1 (BALB/c origin) resulted in cell death with an ED_50_ of 3.94 and 3.78 µM, respectively (Figures 4C,D). To confirm that R11-NLS-pep8-mediated cell death is not restricted only to murine tumors, we tested the effect of R11-NLS-pep8 on MDA-MB-231 human breast adenocarcinoma. Similar to the effect on murine lines, R11-NLS-pep8 induced cell death of MDA-MB-231 cells with an ED_50_ of 4.07 µM (Figure 4E). Cell death of B16, 4T1, and MDA-MB-231 lines was not due to the CPP R11-NLS moiety since treatment with R11-NLS-pep8short did not induce any cell death. A strong induction of cell death was also seen with the positive APIM peptide control, yet to a lesser extent as compared with R11-NLS-pep8. Note that the second positive control of CPT induces lower, but stable levels of cell death, which may result from the short time window (24 h) between the treatment and the cells death recording. It has been reported that the APIM peptide share part of its binding site with PIP box-based peptides, which binds within the IDCL region of PCNA (38). To investigate whether the binding site of NKp44-pep8 is overlapping with the binding site of the APIM peptide, we performed ELISA-based inhibition assay. We studied R11-NLS-pep8 (positive control), R11-NLS-pep8short (negative control) and APIM as inhibitors of pep8 binding to PCNA. All R11-NLS-peptides show a statistically significant change relative to Bio-pep8; however, R11-NLS-pep8 significantly inhibited the binding of pep8 to PCNA by 35%, whereas R11-NLS-pep8short and APIM exhibited inhibition of 10 and 7%, respectively (Figure 4F). Therefore, binding sites of pep8 and APIM are not overlapping.

### R11-NLS-pep8 Injected Systemically Controls Tumor Growth *In Vivo*

**Figure 5:**
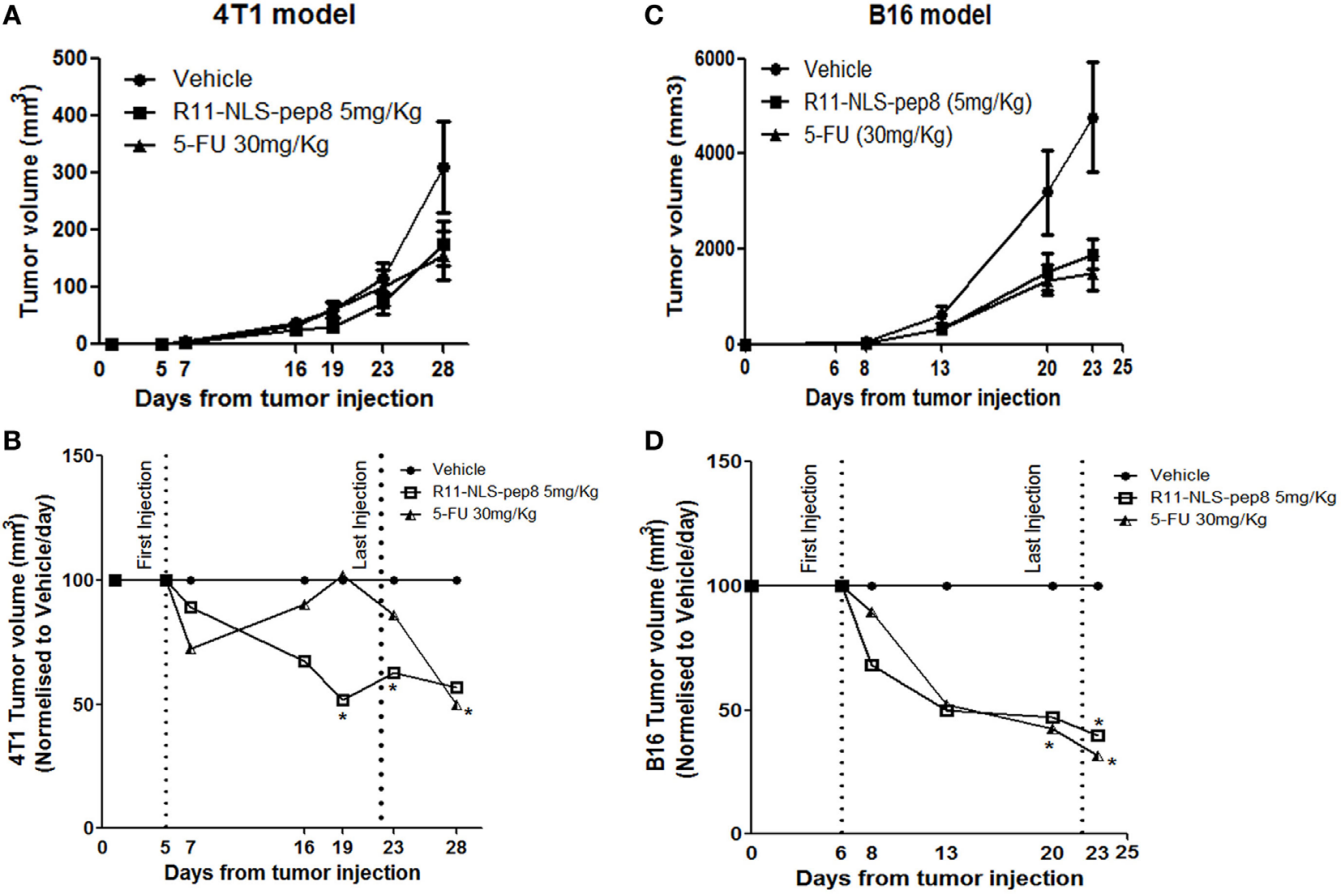
Effect of R11-NLS-pep8 on 4T1 and B16 *in vivo* tumor models. **(A)** 4T1 cells or **(C)** B16 cells were injected into the nipple pad or flank of young BALB/C females or C57BL/6 males, respectively (day 0). At day 5, mice were divided to subgroups according to mice weight. Treatment was started at day 5 (4T1) or day 6 (B16) and end at day 22. Treatment was administrated every 2 days three times per week; 5-FU was administered intraperitoneally (IP) while R11-NLS-pep8 was administrated intravenously (4T1) or IP (B16). Tumor volume (mm^3^) was measured at days as indicated on graphs mean tumor volume (mm^3^), **(B)** 4T1 and **(D)** B16 were normalized to vehicle/day to show the effect of treatment on tumor volume per day. Statistical analysis was performed on original tumor volume data. Unpaired *t*-test; one-tail, *p < 0.05.

In our *in vitro model* R11-NLS-pep8 showed promising results across mouse and human cancer cell lines. Next, we wanted to test whether R11-NLS-pep8 can mediate tumor growth arrest *in vivo*. For the effective R11-NLS-pep8 dose (mg/kg) that can be administered to mice, we studied several concentrations of peptide and different injection routes. For the 4T1 mouse breast cancer we employed IV route of therapy. Figure 5A shows that growth suppression effect of R11-NLS-pep8 in 5 mg/kg was comparable to the effect of 5-FU injected IP at 30 mg/kg dose. For clarity, Figure 5B present the *in vivo* 4T1 results in values normalized to vehicle treatment for each measurement day. Differences between growth in vehicle-treated mice and R11-NLS-pep8-treated mice were statistically significant on days 19 and 23. Note that treatment was stopped on day 22, and measures on day 28 showed a statistically non-significant difference (*p* = 0.1). Growth suppression effect of 5-FU treatment, served as positive control, was clear yet non-significant on days 19 and 23. Interestingly, although the treatment was stopped on day 22, accumulated 5-FU effect was significant on day 28.

For the B16 model, we compared 5-FU and R11-NLS-pep8 inoculated in the same anatomical site (IP), yet peptide was injected at a dose of 5 mg/kg while 5-FU was injected as before at a dose of 30 mg/kg. As with the 4T1 model, 5-FU and peptide treatments were similar in their effect on B16 growth (Figure 5C). For clarity, Figure 5D present the *in vivo* B16 results in values normalized to vehicle treatment for each measurement day. Note that both treatments were stopped on day 22, but measurements on day 23 showed that both treatments significantly suppressed B16 growth.

Initial toxicity studies have been performed with the 5 mg/kg dose injected into mice every 2 days for 3 weeks, and analysis of mouse weight and well-being was meticulously observed during this time. Also, livers, spleens, and lungs were harvested and weight. No significant changes were observed in mice weights and well-being during the experiment and in organs weights at the end of the experiment (data not shown).

As NKp44 is not expressed on mouse NK cells, the anticancer effect of NKp44-pep8 *in vivo* can probably not be attributed to upregulation in mouse NK cell function, which is known to promote tumor rejection.

## Discussion

In this study, we dissected NKp44 binding to PCNA by employing peptide mapping screen. We have identified that NKp44-pep8 (amino acids: 57–76) can specifically bind PCNA and block the binding of NKp44 to the recombinant PCNA protein. NKp44-derived pep10, sharing 10-aa with pep8 (NKp44-pep10 amino acids: 67–86) also interacted with PCNA, indicating this 10-aa sequence is part of the NKp44 binding site to PCNA; yet as independent sequence these 10-aa did not manifest binding to PCNA. Moreover, NKp44-pep8 but not pep10 could block NKp44–PCNA interaction and upregulate NK92 cell function (Figure 1). We therefore employed the whole 20-aa sequence of peptide 8, seeking to test pep8 as a peptide-based agent that can penetrate into cells and target intracellular PCNA.

Proliferating cell nuclear antigen was originally described as an intracellular protein, localized in the cell nucleus. Over the years, PCNA was characterized as a hub protein, interacting with more than fifty intracellular proteins, and with a functional role in DNA replication, cell cycle regulation and apoptosis (13–16). Tumor cells upregulate PCNA levels and PCNA is used as a prognosis marker in various cancers (9, 20–27). Targeting intracellularly PCNA by small molecules or peptides shown promising results as anticancer agent (28, 29). To inhibit PCNA function, PCNA-derived peptides can be used to block PCNA from interacting with its ligands or, alternatively, employing ligands-derived peptides that block the ligands from interacting with PCNA (28, 29). The NKp44-derived peptide that we characterized, belong to the second group but it is entirely different from other published members in this group that their sequence is based on a consensus-binding motifs identified for intracellular protein ligands of PCNA that their binding involve the IDCL region of PCNA. As was shown by others, targeting PCNA using peptides that blocks (i) interactions mediated by the PCNA IDCL domain or (ii) PCNA tyrosine phosphorylation (Y211), can lead to inhibition of cell proliferation or/and promote apoptosis, both *in vitro* and *in vivo* (27, 28, 34). PCNA can be found in the cytosol where it can interacts with procaspases and prevent apoptosis. Targeting PCNA in multiple myeloma cell line using the peptide ATX-101 (38) or in human neutrophils by using a peptide derived from p21 led to apoptosis by blocking the interaction of PCNA with procaspases (58).

Treatment with NKp44-pep8, fused to CPP, led to cancer cell death *in vitro* and suppression of tumor growth *in vivo* (Figures 2–5). These results indicate that NKp44-pep8 target PCNA in a functional domain as the caPep-, Y211F-, PIP-, and APIM-based peptides (25, 27, 28, 34). Moreover, caPep, Y211F, PIP and APIM are all derived from PCNA or from intracellular proteins while NKp44-pep8 is originated from an extracellular immune protein (1, 33, 54) and does not contain the PIP or APIM motifs. This character of the NKp44-derived peptide may indicate that its binding site within PCNA is different from those reported for the APIM/PIP containing peptides. Indeed, it was reported that some PCNA-binding proteins can utilize another binding site on PCNA (18). An experimental support to this assumption is our results showing that APIM peptide cannot block the binding of pep8 to PCNA (Figure 4F).

Utilizing agents against PCNA raise a concern as PCNA is a hub protein that takes part in many cellular interactions also in non-cancer proliferating cells (14, 17). By using the R11-NLS CPP, we were able to show that targeting intracellular PCNA by pep8 can lead to cell death; however, the R11-NLS CPP is not specific to cancer cells and *in vivo* may facilitate peptide penetration to non-cancer cells and harm non-cancer proliferating cells such as activated immune cells. Nevertheless, a few points should be considered when addressing this concern; (1) a therapeutic window, when comparing between cancer vs, normal cells, as PCNA levels are higher in cancer cells (32), (2) vascular permeability of the tumor tissue relative to normal tissue (allowed the tumor environment to absorbed more peptide) and the irregular direction of blood flow in the tumor blood vessels network that will increase the time frame of the peptide at the tumor tissue relative to the normal tissue (59), (3) at the tumor microenvironment, activated immune cells [as neutrophils, macrophages, dendritic cells (MDSC), and Treg] are reprogrammed to support tumor growth. Targeting these cells and cancer cells can be beneficial in some cases (60–64). Altogether, clinical application of peptides or small molecules against PCNA should take this concern into account and try to achieve a more specific drug delivery to target cancer cells.

NKp44 interaction with PCNA is also unique relative to other PCNA interactions, as NKp44 is a receptor that is expressed on the NK cell membrane (1). The interaction of NKp44 with PCNA was shown to have an effect on NK cell function, an effect that is dependent on NKp44 splice variants expression (12). Membrane expression of PCNA was shown to be associated with HLA-I expression on cancer cell lines (7). In particular, the HLA class I histocompatibility antigen, Cw-4 alpha chain was reported to bind PCNA (14, 18). Furthermore, residues 65–79 of the α-chain of HLA-DQA03011 can interact with PCNA and block T cell proliferation (65). The accumulating evidences of PCNA expression on the membrane of cancer cell line and cancer stem cells (8, 10), and the interaction of PCNA with NKp44 and HLA class I or II (7), open the possibility of the existence of PCNA as an immune regulatory protein in the extracellular level. NKp44-pep8 interaction with PCNA (Figure 1), strength the evidence of PCNA role in regulation of the extracellular immune response as NKp44-pep8 target PCNA in a functional domain, which lead to cancer cell death.

Proliferating cell nuclear antigen can be found on the membrane of cancer cells and on exosomes secreted by cancer cells (4, 7, 66). Therefore, the ability of R11-NLS-pep8 to penetrate the cancer cell and interact with intracellular PCNA could potentially be reduced by interaction with cell membrane- or exosome-associated PCNA. Yet, this apparent limitation could benefit the treated patient due to possible enhancement of anticancer immunity; NKp44 expression is associated with NK activation. Since PCNA is an inhibitory ligand for NKp44 (4, 7), it is plausible that treating patients with pep8/CPP-pep8 will lead to increase in the anticancer activity of NKp44+ NK cells by blocking NKp44–PCNA interaction (Figure 1B) (4, 7, 12). In the case of exosomes, it was reported that exosomal PCNA inhibits NK cell function against the prostate cancer cell line DU145 (66). Therefore, CPP-pep8-mediated blocking of exosomal PCNA interaction with NK cells can also lead to increase in NK activity against cancer cells. All of these concern and hypotheses should be confront and tested under controlled conditions.

The NKp44-pep8 site on NKp44 contains the amino acids R47, K53, and R55 which are part of the receptor, single V-type Ig-like domain. These amino acids were shown to take part in the recognition of heparan sulfate (HS) by NKp44, which lead to NK cell activation (67, 68). NKp44 inhibitory effect on NK cell function following the recognition of PCNA is mediated by an ITIM-like sequence found on the NKp44-1 splice variant cytoplasmic domain (4, 12). On the contrary, the NKp44 viral ligand, the influenza virus hemagglutinin, which leads to NK cell activation, was shown to require glycosylation sites located on NKp44 hinge regain (2, 3, 69, 70). This overlapping in the recognition site for HS and PCNA raise the question, does PCNA inhibitory effect on NK cell function is also by blocking other cellular ligands, such as HS? The results also point toward two functional domains on NKp44, (i) the single V-type Ig-like domain and (ii) the hinge domain. However, using peptide screen method has limitations, as it depends on the 3D structure of the peptide relative to the all protein structure (e.g., pep8short). The full binding site of PCNA on NKp44 is yet to be characterized and will help to reveal the complex mechanism that is associated with the NKp44 function.

To summarize, our results show that NKp44-pep8 interact with PCNA in a specific manner and by targeting with intracellular PCNA, can lead to apoptosis of cancer cell lines *in vitro* and tumor growth arrest *in vivo*.

## Ethics Statement

This study was carried out in accordance with the recommendations of “The Institutional Animal Care and Use Committee (IACUC) of Ben-Gurion University of the Negev (BGU).” The protocol was approved by the “The Institutional Animal Care and Use Committee (IACUC) of Ben-Gurion University of the Negev (BGU’s IACUC),” permit: 31.35.13.

## Author Contributions

AP initiated the project; AS, KK, RP, RY, YK, RA, and AP planned experiments; AS, KK, RP, RY, IK, SG, YK, OG-Y, and TR performed and analyzed experiments; AS, KK, AC, RY, and AP further analyzed experiments and wrote the manuscript.

## Conflict of Interest Statement

The authors declare that the research was conducted in the absence of any commercial or financial relationships that could be construed as a potential conflict of interest. The reviewer RP and handling Editor declared their shared affiliation. The reviewer VU declared a shared affiliation, with no collaboration, with one of the authors AC to the handling Editor.
